# Surgical management of reproductive disease in aquarium managed female elasmobranchs

**DOI:** 10.3389/fvets.2026.1958413

**Published:** 2026-10-05

**Authors:** Daniela Yuschenkoff, Lance Adams, Melissa Joblon, Kathryn Tuxbury, Charles Innis, Benjamin Balducchi, Michael McEntire, Natalie D. MyIniczenko

**Affiliations:** 1 Oklahoma City Zoo, Oklahoma City, OK, United States; 2 Aquarium of the Pacific, Long Beach, CA, United States; 3 New England Aquarium, Boston, MA, United States; 4 Biodôme de Montréal, Montréal, QC, Canada; 5 SeaWorld San Antonio, San Antonio, TX, United States; 6 Disney's Animals, Science and Environment, Animal Programs, Lake Buena Vista, FL, United States

**Keywords:** gonad, ovariectomy, ovary, shark, stingray, surgery

## Abstract

**Introduction:**

Reproductive disease in female elasmobranchs has been increasingly reported in the literature, though there are limited descriptions of therapeutic intervention.

**Methods:**

This retrospective case series details the surgical management of 20 female elasmobranchs representing nine species. Data for this study were collected from the medical records and necropsy reports of elasmobranchs at six separate zoos and aquaria.

**Results:**

All animals presented with ovarian or uterine abnormalities, including ovarian enlargement (either due to the presence of cysts and/or chronic follicle retention), yolk coelomitis, and excess uterine fluid. Medical management, primarily hormonal and antibiotic therapy, failed to resolve clinical signs in these cases, and the patients were unlikely to survive long-term. All animals underwent complete or partial unilateral ovariectomy (22 procedures total). Surgical approach, anesthetic protocols, and intraoperative treatments varied by species and institution. Post-operative complications were common, particularly hyporexia, dehiscence, and persistent bacterial coelomitis (when already present prior to surgery). Two Chilean round rays (*Urotrygon chilensis*) required a second ovariectomy after a functional contralateral ovary developed post-unilateral ovariectomy. In this group, 32% of the patients experienced post-operative mortality directly due to surgical or post-surgical complications; 18% died post-operatively due to pre-existing conditions or systemic compromise. However, this was improved with balanced anesthetic protocols and more aggressive peri-procedural supportive care based on the authors’ experience.

**Discussion:**

In successful procedures, ovariectomy led to the resolution of clinical signs, demonstrating that ovariectomy is a viable therapeutic option for reproductive disease in elasmobranchs, though early intervention, post-operative monitoring, and supportive care are recommended.

## 1 Introduction

Reproductive disease is increasingly diagnosed in managed elasmobranch populations, though there is limited literature regarding diagnosis, management, and treatment. However, more recent case reports have documented cystic ovarian disease in cownose rays (*Rhinoptera bonasus*), follicular stasis and cystic ovaries in southern stingrays (*Hypanus americanus*), mucometra in southern stingrays, and egg retention in sand tiger sharks (*Carcharias taurus*) (1–4). In advanced stages, these diseases can result in significant morbidity and are likely to be fatal if left untreated (2–4).

The anatomy and physiology of the female reproductive tract vary widely among elasmobranchs. Ovarian size, number, and location will differ based on species, reproductive strategy, and fecundity. Myliobatiformes exhibit aplacental viviparity and may possess either single or paired ovaries and uteri (5, 6). Cownose rays, southern stingrays, and potamotrygonids typically exhibit a primary functional left-sided ovary with a functional left uterine horn, though right-sided pregnancies have been reported in cownose rays (5–13). A dominant right ovary, a non-functional left ovary, and bilaterally functional uterine horns have been infrequently identified in some Urotrygonidae species (6, 14–16). Conversely, bamboo sharks (*Chiloscyllium* spp.) and epaulette sharks (*Hemiscyllium ocellatum*) have a single right-sided ovary and exhibit oviparity with two functional uterine horns (5, 17–21). Similarly, members of the Carcharhiniformes family typically have a single right ovary, though a vestigial left ovary has been noted in some species (22). The epigonal organs are the primary lymphomyeloid tissue in elasmobranchs and are typically adjacent to or surrounding the ovarian tissue, but this is also species variable, and in some species, the ovary is intermixed with the epigonal (5, 6, 11, 12, 22).

Due to the paucity of clinical reports and the anatomical diversity in these taxa, treatment options for reproductive disease have not been well established. Hormonal treatment can be considered but appears to have an inconsistent response and variable efficacy, which may be due to the severity of the disease and physiologic hormonal dysregulation (reproductive or thyroidal) (23, 24). Ovariectomy may be a curative surgical option for elasmobranchs with ovarian and some uterine diseases. In healthy southern stingrays, ovariectomy has been primarily utilized as a contraceptive technique (2, 7, 10, 25). Similarly, left ovariectomies were performed in a group of *Potamotrygon* rays to control reproduction (26). Additionally, a laparoscopic ovariectomy procedure has been described in Atlantic stingray (*Hypanus sabinus*) cadavers, though this has not been reported in clinical cases (27).

These surgical reports are limited to healthy or deceased elasmobranchs, and to date, no published reports have documented ovariectomies in female sharks or rays with reproductive disease. This report aims to describe the clinical presentation, surgical procedure, and post-operative outcomes associated with ovariectomy in multiple elasmobranch species with varying degrees of ovarian and/or uterine pathology.

## 2 Materials and methods

### 2.1 Animals and veterinary reports

Data for this study were collected from the medical records and necropsy reports of elasmobranchs at six separate zoos and aquaria. Elasmobranchs met the inclusion criteria if they had a confirmed diagnosis of reproductive disease that was treated surgically, with either a partial or total ovariectomy. Diagnostic approaches varied by case and included ultrasonography, computed tomography, complete blood counts, plasma biochemistry panels, plasma hormone and iodine testing, endoscopy, histopathology, cytology, and bacterial and fungal culture.

Relevant information from the medical records were entered into Microsoft Excel (version 2,408) and included the following parameters: individual identification, institution, species, age (if known), clinical signs, anesthetic drugs and dosage, surgical approach and technique, closure method, perioperative analgesia, antibiotics, fluid therapy, post-operative care, post-operative complications, and histopathologic findings of surgically removed tissue, if available.

## 3 Results

### 3.1 Animals

Twenty elasmobranchs from six separate zoos or aquaria met the inclusion criteria. Table 1 summarizes the demographic data of the studied elasmobranchs, including species and age at time of surgery (if known).

**Table 1:** Demographic data of the studied elasmobranchs, including species and age at time of ovariectomy (if known).

Common name	Species	Age (years)
Brown-banded bamboo shark	*Chiloscyllium punctatum*	>8
Brown-banded bamboo shark	*Chiloscyllium punctatum*	10
Chilean round ray	*Urotrygon chilensis*	5
Chilean round ray	*Urotrygon chilensis*	4, 5 (two surgeries)
Chilean round ray	*Urotrygon chilensis*	4, 6 (two surgeries)
Cownose ray	*Rhinoptera bonasus*	>10
Cownose ray	*Rhinoptera bonasus*	>27
Cownose ray	*Rhinoptera bonasus*	>3
Coral cat shark	*Atelomycterus marmoratus*	11
Epaulette shark	*Hemiscyllium ocellatum*	10
Haller’s round ray	*Urobatis halleri*	>9
Southern red stingray	*Hypanus americanus*	6
White-blotched river stingray	*Potamotrygon leopoldi*	7
White-spotted bamboo shark	*Chiloscyllium plagiosum*	>8
White-spotted bamboo shark	*Chiloscyllium plagiosum*	>9
White-spotted bamboo shark	*Chiloscyllium plagiosum*	>10
White-spotted bamboo shark	*Chiloscyllium plagiosum*	>10
White-spotted bamboo shark	*Chiloscyllium plagiosum*	13
White-spotted bamboo shark	*Chiloscyllium plagiosum*	>14
White-spotted bamboo shark	*Chiloscyllium plagiosum*	28

The mean age ± SD of the study population was 11 ± 6 years. Nine species were included in this study, with the most common being the white-spotted bamboo shark (*Chiloscyllium plagiosum*, 7 animals), followed by Chilean round ray (*Urotrygon chilensis*, 3 animals), cownose ray (*Rhinoptera bonasus*, 3 animals), brown-banded bamboo shark (*Chiloscyllium punctatum*, 2 animals), and one case involving a coral cat shark (*Atelomycterus marmoratus*), an epaulette shark (*Hemiscyllium ocellatum*), a Haller’s round ray (*Urobatis halleri*), a southern red stingray (*Hypanus americanus*), and a white-blotched river stingray (*Potamotrygon leopoldi*). In total, there were 22 surgical reports, as two Chilean round rays had two separate ovariectomy procedures, as a functional right ovary developed after removal of the left ovary.

### 3.2 Clinical indications for surgical intervention

The initial reported clinical signs in these cases were often nonspecific. The most commonly recognized clinical signs included coelomic distension (12 cases, 8 of which were due to coelomic effusion), hyporexia or anorexia (9 cases), lethargy (4 cases), and yolk leaking from coelomic pores (3 cases). In six cases, reproductive abnormalities were first noted on routine ultrasound evaluation. The time from first clinical signs or diagnosis of reproductive disease to surgery ranged from 0.3 to 132 months, with an average time of 21 months.

Coelomic ultrasound was utilized to diagnose reproductive abnormalities in all of the reported cases, and endoscopy (either coelioscopy through a cloacal pore or cloacoscopy) was used in nine cases. Computed tomography (CT) with contrast was utilized in four cases with an iohexol dose ranging from 380 to 700 mg/kg IV (Figure 1). The primary reproductive abnormalities are listed in Table 2. Ovaries containing cysts, follicular retention, and variably-sized, irregularly shaped follicles were the most common finding, noted in 12 cases. All of the Myliobatiforms in this report presented with these ovarian changes, and two Chilean round rays presented with this issue twice, after the right ovary developed disease following unilateral ovariectomy. In addition to exhibiting abnormal ovaries, the southern red stingray and all three Chilean round rays in this report exhibited a fluid-filled uterus. Two of these Chilean round rays presented with this abnormality twice, and two of the cases were due to bacterial metritis. A single brown-banded bamboo shark also presented with a cystic ovary and was the only shark to exhibit this abnormality in this report.

**Figure 1:**
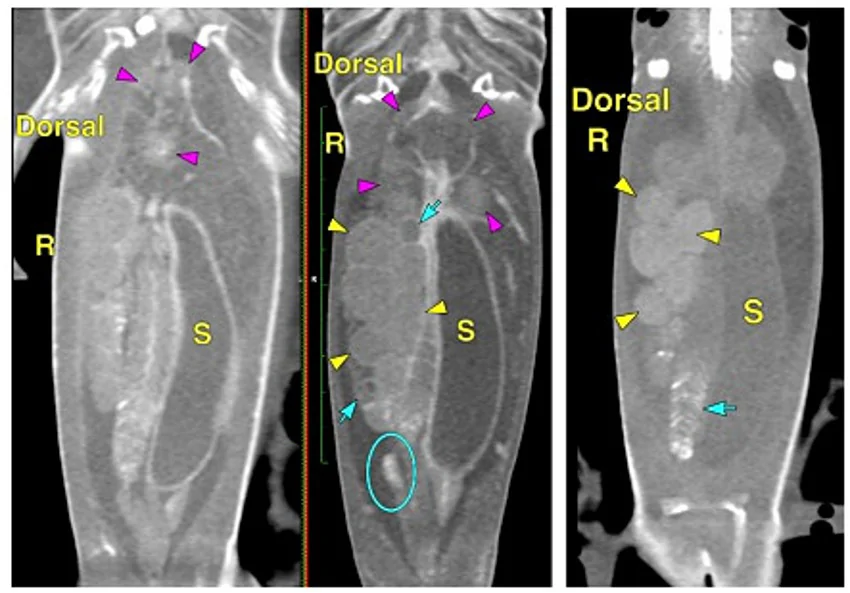
Pre-operative computed tomography with contrast in a coral cat shark. There are multiple variably sized follicles/oocytes in the region of the right ovary with a few pinpoint mineral foci (arrowheads). No follicles/oocytes are identified within the oviduct/uterus. There is a focal, mildly thickened region within the distal aspect of the oviduct with strong contrast enhancement (middle image, oval). Image courtesy of David Reese, DVM, DACVR, MRCVS (VetCT).

**Table 2:** Reproductive abnormalities reported in elasmobranchs before surgical intervention, listed by affected species.

Reproductive abnormalities	Number of instances
**Ovarian cysts/follicular enlargement**	12
Chilean round ray	5
Cownose ray	3
Haller’s round ray	1
Brown-banded bamboo shark	1
Southern red stingray	1
White-blotched river stingray	1
**Yolk coelomitis**	9
White-spotted bamboo shark	7
Brown banded-bamboo shark	1
Coral cat shark	1
**Ovarian enlargement (non-cystic)**	4
White-spotted bamboo shark	3
Coral cat shark	1
**Fluid-filled uterus**	6
Chilean round ray	5
Southern red stingray	1
**Irregular oviposition**	4
Epaulette shark	1
Brown-banded bamboo shark	1
White-spotted bamboo shark	1
Coral cat shark	1
**Metritis**	2
Chilean round ray	1
Southern red stingray	1
Ovarian follicle rupture	1
White-spotted bamboo shark	1

Yolk coelomitis was noted in a brown-banded bamboo shark, a coral cat shark, and all seven white-spotted bamboo sharks in this case series (Figure 2). Non-cystic ovarian enlargement was seen in four of these sharks. One white-spotted bamboo shark had a ruptured ovarian follicle, which was the main clinical indication for surgical intervention. Irregular oviposition, occasionally accompanied by intermittent yolkless egg cases and persistent yet erratic bouts of egg-binding, was seen in one brown-banded bamboo shark, an epaulette shark, a coral cat shark, and one white-spotted bamboo shark.

**Figure 2:**
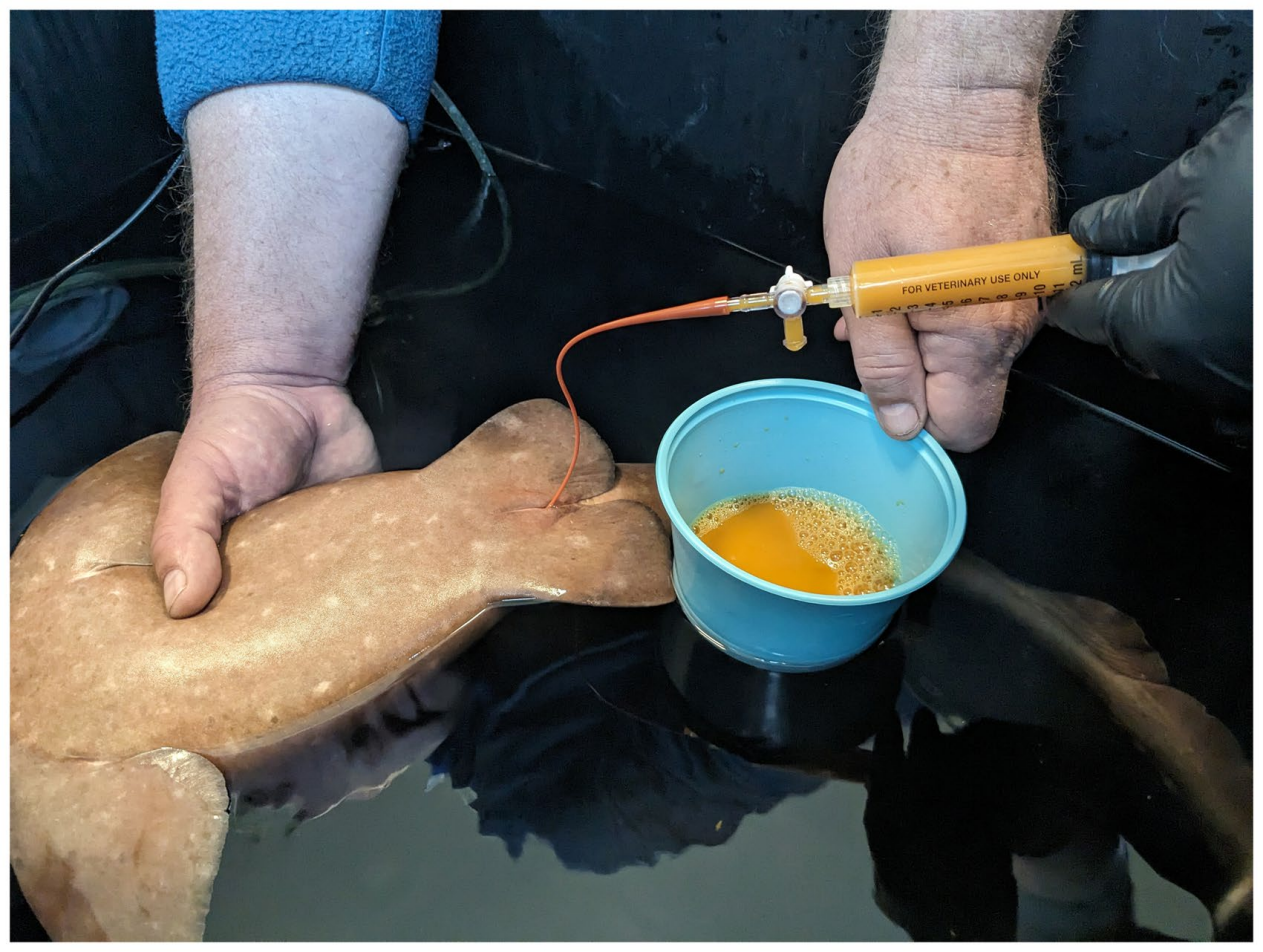
A white-spotted bamboo shark (*Chiloscyllium plagiosum*) with coelomic distension and effusion characteristic of yolk coelomitis. The coelomic fluid is being aspirated through a coelomic pore. The coelomic fluid can vary in color and opacity.

Plasma hormones were measured in 10 cases. Seven of these elasmobranchs had plasma concentrations of estradiol >1,000 pg./mL shortly before or on the day of surgery. Additionally, plasma iodine concentrations were determined in eight animals. Four of these animals evaluated had a total plasma iodine concentration of >400 ng/mL within 6 months preceding ovariectomy, while one animal had concentrations of <25 ng/mL 5 months before surgery.

Before surgical intervention, some more conservative treatments were attempted, but ultimately did not lead to clinical resolution in these cases. Fourteen animals received at least one course of antibiotic therapy preceding surgical intervention, primarily due to coelomitis or metritis. Eight animals received hormonal therapy; these treatments included medroxyprogesterone acetate injections (5 cases), administration of gonadotropin-releasing hormone (GnRH) agonists (Cystorelin) with prostaglandin F2α (PGF2α, Lutalyse) (4 cases), GnRH agonist implants (Deslorelin) (4 cases), GnRH analog injections (3 cases), and oral norethindrone/estradiol (1 case). Seven cases with coelomic effusion or yolk leakage were treated with coelomic flushes, and two sharks with recurrent egg binding were anesthetized for manual egg removal. Though occasional mild improvements were seen with some hormonal treatments (e.g., a reduction in ovarian size with PGF2α), ultimately, none of these less-invasive treatments led to long-term, complete resolution of reproductive disease, so surgical intervention was elected.

### 3.3 Surgical procedure

All animals were anesthetized for ovariectomy and were induced with buffered tricaine methanesulfonate (MS-222) (16 cases), eugenol (AquiS20e) (5 cases), or propofol (1 case). The induction dose of MS-222 varied from 60 to 110 ppm, while eugenol 30 to 60 ppm was used for induction. All animals were maintained with MS-222, with doses ranging from 50 to 95 ppm. Supplemental butorphanol was given intravenously (IV) in 14 cases, and a propofol constant rate infusion (CRI) with intermittent boluses was utilized in 1 case.

Surgical approach varied by taxa, with a dorsal left or right paramedian incision utilized in Myliobatiformes and a right flank or ventral paramedian approach used in Orectolobiformes and Carcharhiniformes (Figure 3). The method of ovarian vessel ligation varied: ligation/cautery device (10 cases), hemoclips and suture (5 cases), hemoclips (4 cases), suture (2 cases). In one case, a partial ovariectomy was performed via blunt dissection. The epigonal organ was completely removed in 15 cases, including two Chilean round rays with bilateral epigonal removal, while the epigonal organ remained in the remaining cases (Figure 4). The incision was closed with a two- or three-layer closure in all cases (Figure 5). The skin was closed with a variety of suture materials: poliglecaprone (11 cases), polydioxanone (5 cases), nylon (3 cases), and polypropylene (2 cases). Skin staples were utilized in a single case.

**Figure 3:**
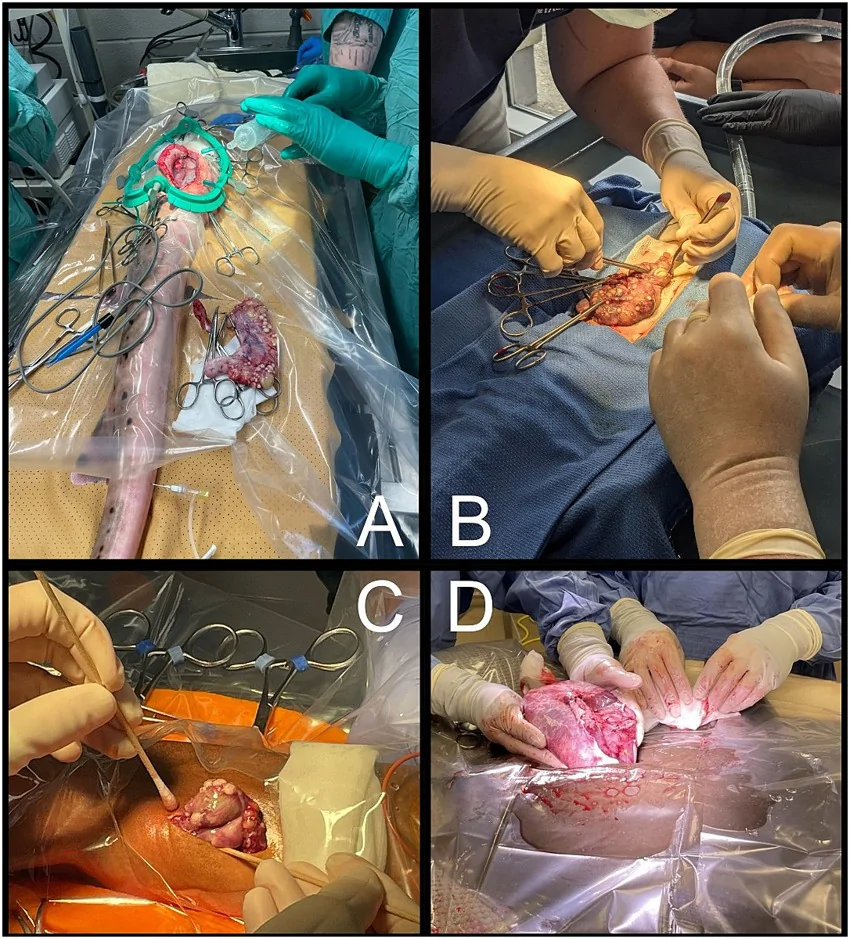
Intraoperative images from an epaulette shark **(A)**, white-spotted bamboo shark **(B)**, brown-banded bamboo shark **(C)**, and cownose ray **(D)** undergoing an ovariectomy. **(A)** The ovary has been exteriorized from a right flank incision. **(B)** The ovary has been completely excised from a right flank incision. **(C)** The ovary has been partially exteriorized from a right paramedian incision. **(D)** The ovary has been partially exteriorized from a left dorsal paramedian incision.

**Figure 4:**
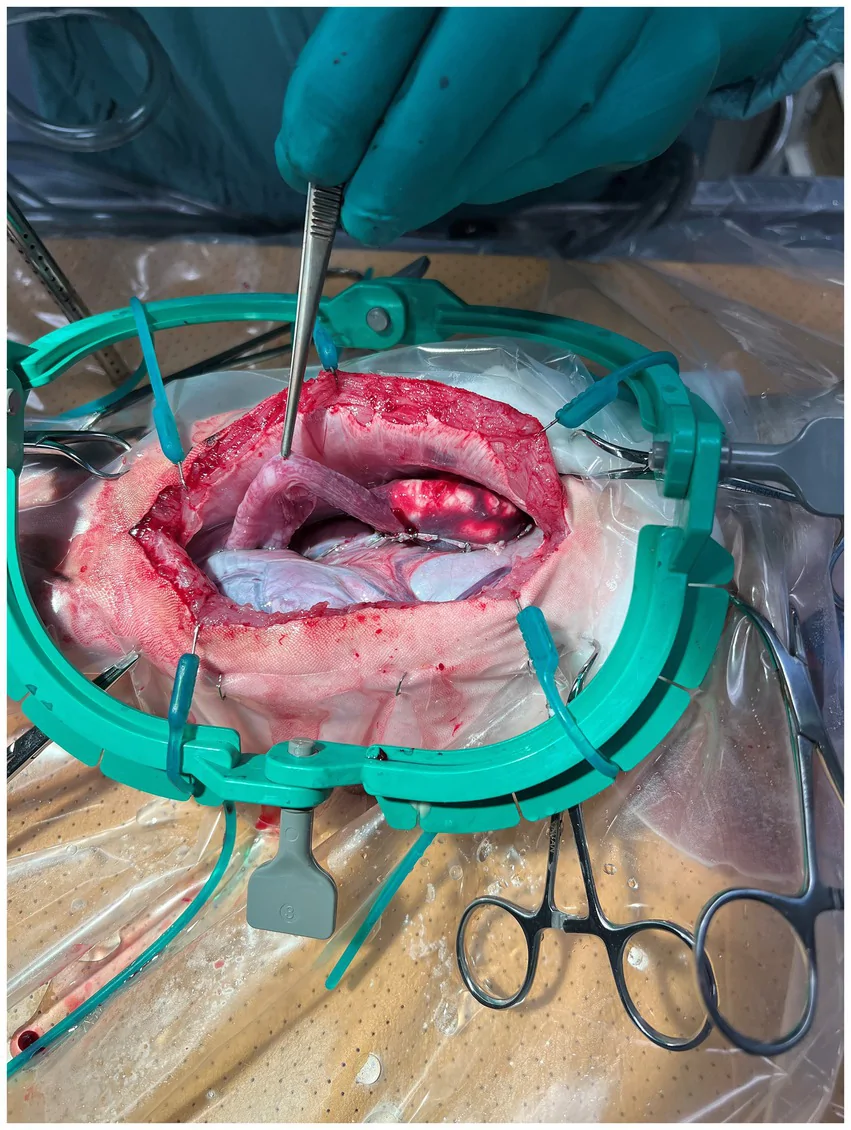
Intraoperative image of an epaulette shark after the ovary has been excised. The uterus and epigonal organ remain.

**Figure 5:**
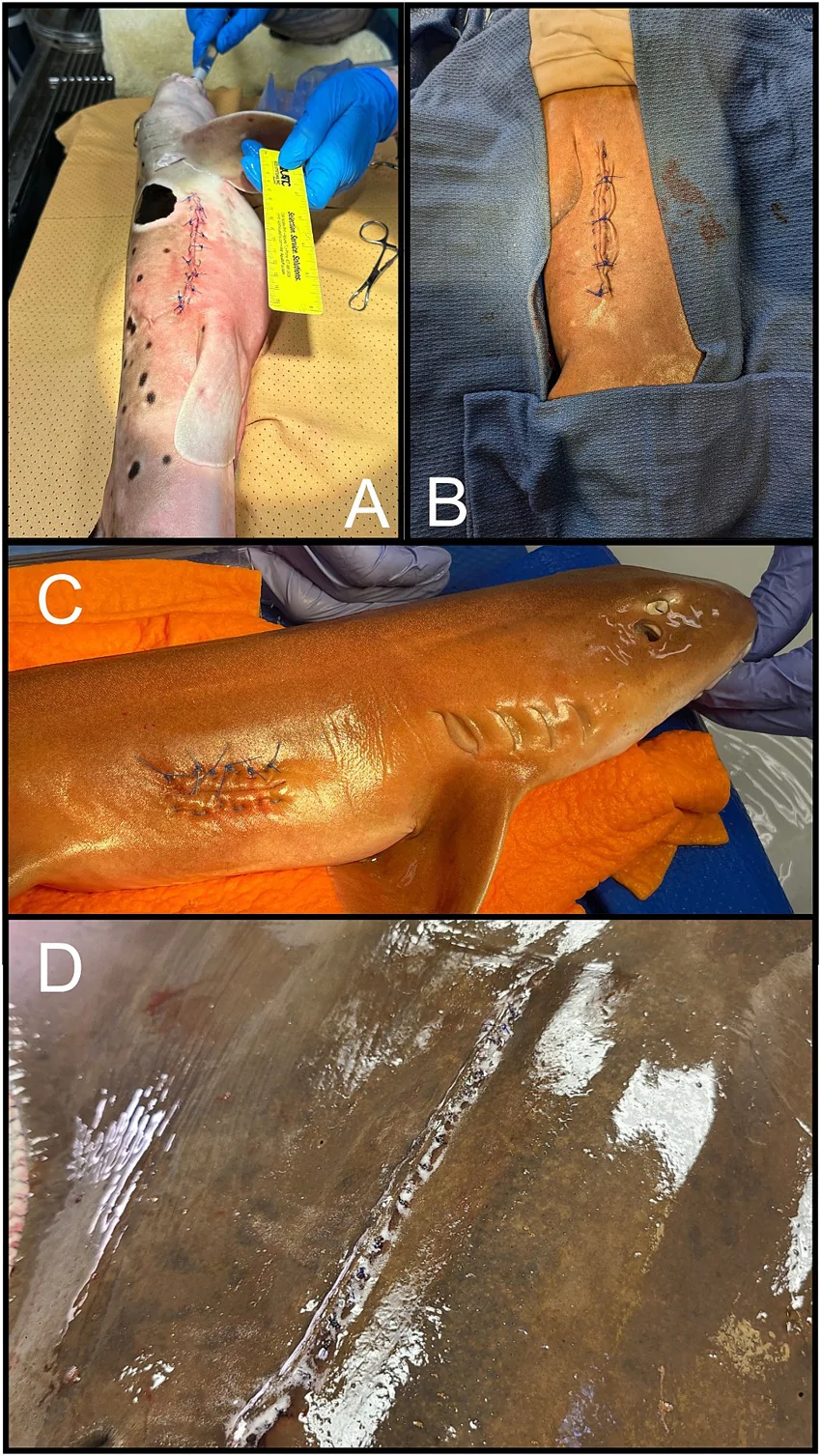
Post-operative images of skin incision closures of an epaulette shark **(A)**, white-spotted bamboo shark **(B)**, brown-banded bamboo shark **(C)**, and a cownose ray **(D)**. **(A)** Right flank incision in an epaulette shark closed in a horizontal mattress suture pattern. **(B)** Right paramedian incision in a white-spotted bamboo shark sutured in a horizontal mattress pattern. **(C)** Right paramedian incision in a brown-banded bamboo shark sutured in an alternating horizontal/vertical mattress pattern. **(D)** Left dorsal paramedian incision in a cownose ray closed with a vertical mattress suture pattern.

All patients, except for one (due to concerns for hemostasis), received intra-operative analgesia and antibiotics, while 14 received IV fluids (Table 3). As analgesia, ketoprofen was most commonly utilized, but meloxicam, hydromorphone, or other NSAIDs were also occasionally used. Ceftazidime was the most frequently administered antibiotic, followed closely by danofloxacin, then florfenicol, amikacin, and ceftiofur. Elasmobranch balanced salt solution (elasmobranch “Ringer’s”) was given intravenously in 12 cases, given with Intralipid (B. Braun Medical Inc., Bethlehem, PA) in 10 cases, while Hank’s Balanced Salt Solution (Sigma-Aldrich, Missouri, USA) with Dextrose 50%, Lactated Ringer’s Solution, and an allogenic blood transfusion were each used in one case.

**Table 3:** Intra-operative administration of analgesics, antibiotics, and intravenous fluids listed by the number of elasmobranch surgical cases treated.

Intra-operative treatment	Number of cases treated
**Analgesia**	21
Ketoprofen	15
Meloxicam	4
Hydromorphone	3
Robenacoxib	2
Carprofen	1
**Antibiotics**	19
Ceftazidime	9
Danofloxacin	8
Florfenicol	5
Amikacin	4
Ceftiofur	4
**Intravenous fluids**	14
Elasmobranch ringers	12
Intralipid	10
Hank’s BSS + dextrose 50%	1
Lactated Ringer’s Solution	1
Allogenic blood transfusion	1

### 3.4 Post-operative care and complications

Post-operative care varied widely by institution and clinical status of the patient after ovariectomy. Commonly utilized treatments included continued analgesic therapy (16 cases), continued antibiotic therapy (13 cases), assisted feeding (10 cases), IV fluids (8 cases), and IV Intralipid (6 cases). Coelomic flushes via the coelomic pore were utilized in cases with post-operative coelomitis and effusion (7 cases). Patients exhibiting post-operative anemia or hemorrhage were often administered injectable Vitamin K (3 cases), and Epoetin alfa was used in a single case.

Post-operative complications were noted in most cases and ranged in severity (see Table 4). The most common complications reported were anorexia or hyporexia (11 cases) and post-operative death directly related to the procedure (7 cases). All of the post-operative mortalities were found deceased 3–10 days after surgery; one was found deceased the same night, and two were euthanized the same day due to prolonged apnea/anesthetic recovery or significant intra-coelomic hemorrhage. Death was most commonly due to hemorrhage or incisional infection, but more chronic, preexisting conditions were also the cause of four additional deaths: chronic inanition in three cases, and cardiac disease in one case. Dehiscence was noted in this cohort (4 cases), though this was generally not an immediate post-operative finding. In these cases, dehiscence was initially diagnosed 8 days to 1.5 months after surgery, with continued dehiscence noted 4 months post-operatively in one case. Surgical revision was necessary in three of the cases where dehiscence was noted. Bacterial coelomitis was seen in four cases; two of these cases had pre-existing coelomitis prior to surgery, and two cases (epaulette shark, white-spotted bamboo shark) died within 10 days post-operatively.

**Table 4:** Post-operative complications observed in elasmobranchs following ovariectomy.

Post-operative complications	Number of instances
Anorexia/hyporexia	11
Death/Euthanasia	7
Hemorrhage	3
Incisional infection	2
Gastric perforation	1
Prolonged anesthetic recovery	1
Dehiscence	4
Coelomitis	4 (2 were preexisting)
Anemia	2
Hemorrhage	1

Though post-operative complications were commonly noted, complete or partial removal of the functional, abnormal ovarian tissue led to the resolution of associated reproductive disease in all nine surviving elasmobranchs.

Ovarian tissues were submitted for histopathological evaluation in 18 cases. The most common findings (frequently coexisting) were follicular atresia or regression (*n* = 9), ovarian granulomas (*n* = 5), oophoritis (*n* = 4), fungal endometritis (*n* = 1), ovarian mineralization (*n* = 2), and ovarian glandular neoplasia (*n* = 1). The Haller’s round ray was euthanized due to an unrelated illness, and active folliculogenesis was noted in the remaining right ovary (Figure 6).

**Figure 6:**
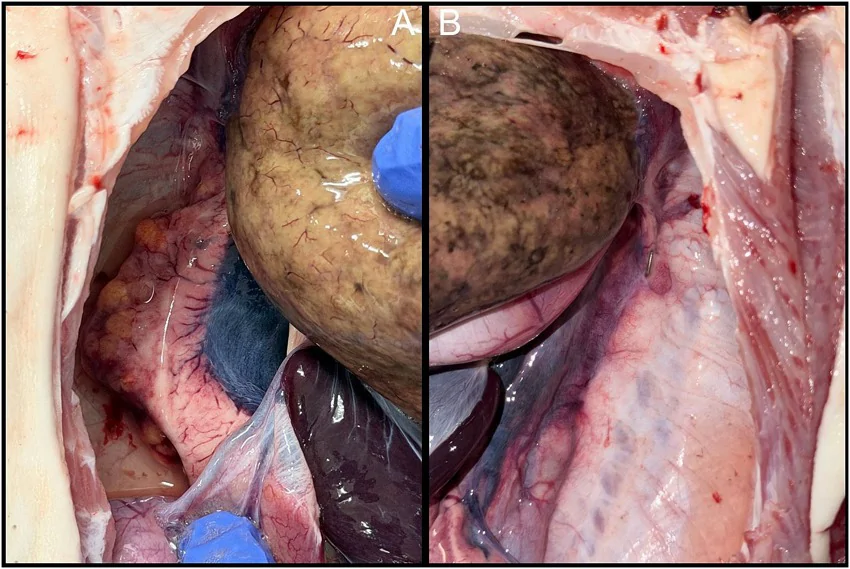
Post-mortem images from a Haller’s round ray showing the right **(A)** and left **(B)** sides of the coelom. This ray was euthanized for an unrelated condition over 4 years after ovariectomy. **(A)** The right ovary is present, and active folliculogenesis was noted on histopathologic review of these tissues. **(B)** Hemoclip noted at the previous ovariectomy site.

## 4 Discussion

Ovariectomy is a viable treatment option for reproductive disease in elasmobranchs. Though surgical intervention is not without risk, based on the cases described in this report, ovariectomy appears to provide long-term, complete resolution for rays and sharks with ovarian and/or uterine disease. It is the opinion of the authors that reproductive disease is an ultimately fatal disease in elasmobranchs. Advanced ovarian disease, as described in cownose rays and southern stingrays, has the potential to cause anorexia and lethargy simply due to the space-occupying effect of the enlarged tissues, preventing expansion of the stomach (1, 3, 4). Additionally, as reproductive tissues can be fragile, tearing can occur when ovaries reach a large size, resulting in fatal hemorrhage or yolk leakage and coelomitis (2). As evidenced by the protracted duration of clinical signs noted in this cohort, reproductive disease in elasmobranchs is often an insidious, chronic disease, and the prolonged average time to surgical intervention (21 months) may have been a contributing factor to the high mortality rate noted in this cohort. Early diagnosis and treatment of reproductive disease may mitigate the sequelae associated with severe disease and improve survival.

In the described cases, ultrasonography was uniformly used to diagnose reproductive abnormalities and assess response to therapy. The utility of routine ultrasounds should be considered as an integral element of an elasmobranch preventative medicine program. Cloacoscopy and/or coelioscopy via the coelomic (“abdominal”) pores was also frequently utilized to further characterize abnormalities in this cohort and represents another minimally invasive diagnostic tool for the diagnosis of reproductive disease in elasmobranchs. Cloacoscopy and subsequent hysteroscopy allow for visualization and sampling of the endometrium, while coelioscopy can facilitate direct visualization of the ovary, the epigonal organ, the liver, and coelomic fluid to assess for potential yolk contamination. Computed tomography with contrast was utilized in four cases in this series and served as a valuable tool to confirm the presence and extent of reproductive disease.

Elevated estradiol was frequently noted in this cohort when evaluated, which is consistent with findings in other elasmobranchs with reproductive abnormalities (1). In wild and aquarium-housed southern stingrays, ovarian and uterine disease were significantly associated with elevated plasma estradiol concentrations (1). Additionally, total plasma iodine levels of <25 ng/mL and >400 ng/mL were seen in several animals in this cohort. In a recent report in southern stingrays, total iodine levels of >400 ng/mL were associated with a negative health status, and a small subset of these animals had reproductive disease and abnormally high plasma iodine (>2000 ng/mL) and follicular iodine (9,000–10,000 ng/mL) (28). Iodine deficiency is the primary cause of thyroid hyperplasia (goiter) in fish (29). In elasmobranchs, thyroid function, and thus iodine concentrations, are intricately tied to reproduction. Thyroid hormone levels increase during egg development and vitellogenesis, thyroidectomy inhibits gonadal development during breeding season in lesser spotted dogfish (*Scyliorhinus canicula*), and circulating T_3_ concentrations are elevated in Atlantic stingrays (*Hypanus sabinus*) during oogenesis, ovulation, and gestation (30). Persistent elevations in iodine were noted early in the disease course of many of the patients in this report, potentially resulting in hormonal dysregulation and further exacerbating reproductive disease. Conversely, iodine levels may not be elevated in the later stages of disease, particularly if patients are not consuming food (and thus supplements) regularly. Given the significant elevations seen in the case reported here, plasma estradiol and total iodine testing should be considered in elasmobranchs with reproductive disease. However, further research is necessary to discern the correlation between thyroid and reproductive disease and to determine if the resolution of reproductive disease results in a return to normal estradiol and iodine plasma values.

Some species-specific predilections were present in this cohort. All Myliobatiformes presented with ovarian disease, which is consistent with previous reports of this finding in cownose rays and southern stingrays (1–4, 25). These abnormal ovaries typically exhibited cystic changes or follicular stasis, characterized by the presence of numerous layered follicles. These changes can be challenging for inexperienced clinicians to recognize, making routine imaging of normal patients invaluable for establishing a baseline of ovarian health. Every white-spotted bamboo shark in this case series exhibited yolk coelomitis as the primary indication of reproductive disease. Chronic egg retention was noted in the epaulette shark and the brown-banded bamboo shark in this report, consistent with the description of a previous report of fatal dystocia in an epaulette shark (31). These cases typically exhibited anorexia, coelomic distension, or lack of egg case passage, and patients required medical intervention for the passage of egg cases over a span of multiple years. Additionally, ovarian plasticity appears to be present in some species, as the Chilean round rays in this cohort developed functional right ovaries after left-ovariectomy, and a functional right ovary was noted in the Haller’s round ray on post-mortem evaluation years after the initial surgery. A similar phenomenon has been noted in *Potamotrygon* spp. rays, after offspring were produced post-unilateral ovariectomy (26). This variance in clinical presentation among taxa likely reflects the wide diversity of reproductive anatomy and physiology throughout elasmobranchs and the importance of understanding normal species-specific anatomy, making it challenging to make generalized recommendations for ovariectomy protocols.

All elasmobranchs in this report were anesthetized using anesthetics commonly utilized in cartilaginous fishes (32–35). Drug selection and dosage were largely dependent on patient species, length of procedure, and clinician preference. In the authors’ experience, supplementing an immersion anesthetic protocol with butorphanol IV led to a smoother anesthetic procedure and conceivably offered additional analgesic coverage.

Previous reports of ovariectomy in elasmobranchs are limited to describing its utility as a contraceptive method or a prophylactic measure to prevent future reproductive disease (2, 7, 10, 25, 26). In these previous reports, the size of the ovary is noted to be one of the major surgical challenges, as very small ovaries may be difficult to distinguish from surrounding epigonal tissue, and very large ovaries may be difficult to remove due to their bulkiness and heavy vascularized tissue (3, 10) (Figure 7). The enlarged ovaries present in many of the Myliobatiform surgeries in this report often required large skin incisions and resulted in poor visualization throughout the surgery. In these cases, the use of a ligation/cautery device was invaluable for minimizing surgical time, obtaining appropriate surgical access, and safely ligating the ovarian vessels. Removal of these large organs may also contribute to acute hypovolemia, with surgical excision causing a rapid decrease in overall blood volume. Additionally, the overall diminutive size of some Myliobatiformes did present some surgical difficulties in the cases reported here, and surgical intervention in smaller animals should be attempted with careful planning and appropriate instrumentation. Regardless of the size of the animal or its ovaries, if the animal is in good nutritional status, a large liver can hinder visualization and removal of the ovaries, even with a dorsal approach. Hysterectomy was not performed in any of the reported cases. Surgical removal of the uterus is unlikely to resolve hormonally driven diseases, and it is a technically challenging procedure due to difficult hemostasis and the proximity of renal structures.

**Figure 7:**
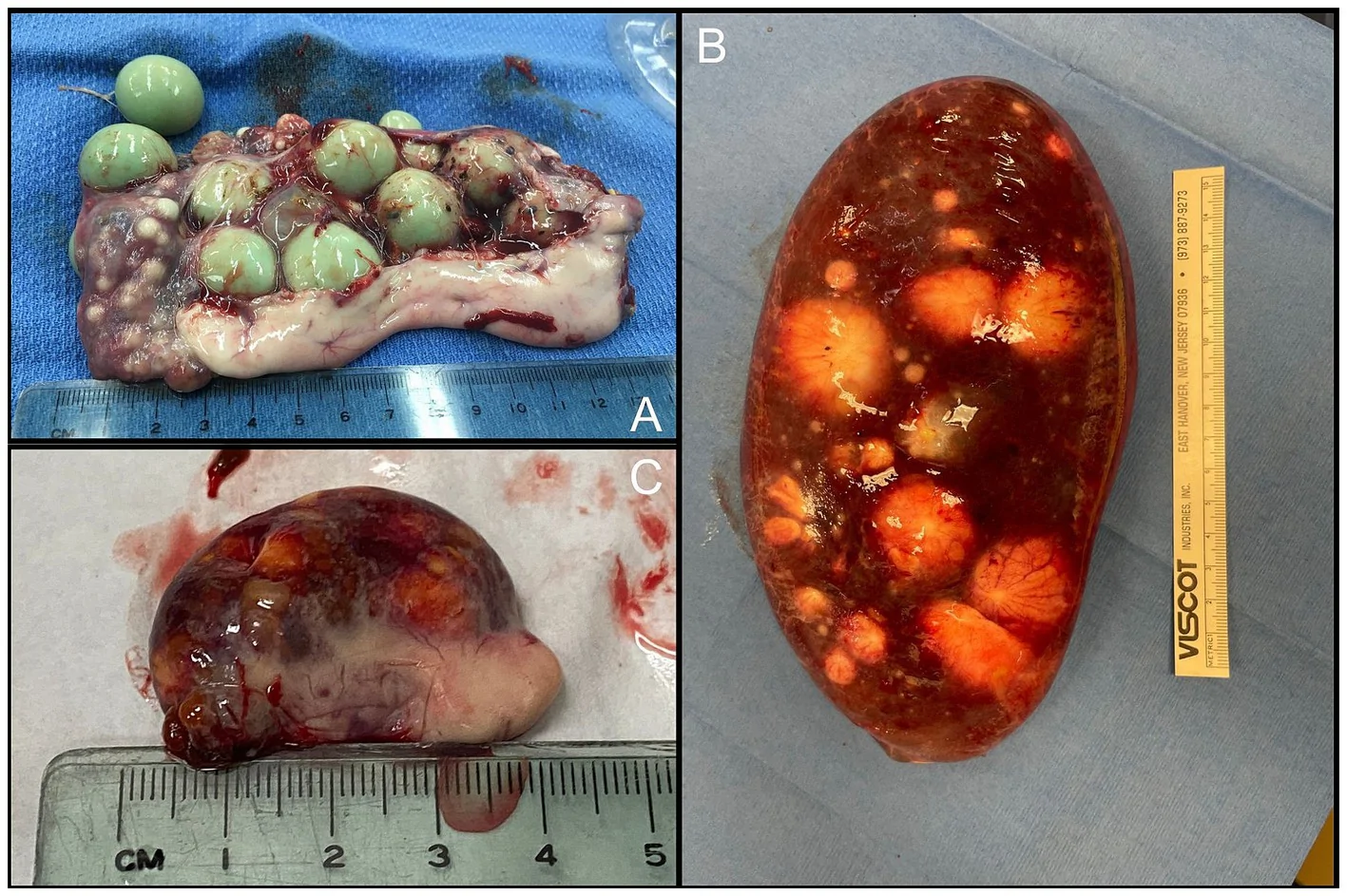
Surgically excised ovaries from a white-spotted bamboo shark **(A)**, a cownose ray **(B)**, and a Chilean ray **(C)**. **(A)** Follicles of various sizes can be seen on this ovary, and the epigonal organ is in close association with ovarian tissue. **(B)** Multiple follicles and cysts can be seen in this cownose ray ovary that was removed *en bloc*. **(C)** The epigonal organ expands into the ovarian tissues of this Chilean ray ovary.

In the reported surgical cases, the ovarian ligation technique and surgical closure method varied with patient size, clinician preference, and institution. Though no deaths occurred in the animals with non-absorbable sutures used for skin closure, this is unlikely to indicate a true association with survival. Over time, external sutures may degrade slowly with exposure to salt water. Based on the experience of the authors, some absorbable sutures do not dissolve internally in elasmobranchs, though they may degrade and become brittle with time. These can be seen years later on post-mortem evaluation. The longevity of suture material inside the body may be a nidus for inflammation and fibrosis. In the authors’ experience, rinsing the suture before use may decrease suture site reactions presumed due to elasmobranch sensitivity to the volatile components in the lubrication that are used for suture storage (see individual Materials Safety and Data Sheets) (36). The surgical procedure in the reported cases was similar to previously reported ovariectomy protocols, though in all previous reports, the elasmobranchs did not exhibit reproductive abnormalities (2, 7, 10, 25, 26). After removal of the ovary, a complete evaluation of the coelomic cavity is recommended to screen for any remnant egg cases and assess the liver to gauge overall nutritional status.

In most of these prior reports, the epigonal organ was completely or partially removed with ovarian tissue (2, 7, 10, 25). The epigonal organ is one of the primary lymphomyeloid tissues of elasmobranchs (5). The epigonal organ can vary widely in its structure and location amongst elasmobranchs, so a careful study of species-specific anatomy is recommended prior to ovariectomy (22). In the present report, the epigonal organ was removed in 15 of the 22 cases, which may reflect the difficulty in dissecting the epigonal organ from the ovary in the presence of reproductive disease. Based on the authors’ experiences, it is often preferable to remove the epigonal organ *en bloc* with the ovary, due to its intimate association with the ovary and its proximity to the vena cava. The epigonal organ can be difficult to separate and retain as it is highly vascularized, and it can be difficult to obtain appropriate surgical visualization and access. Additionally, the epigonal organ is very fragile and is not amenable to cautery, retention of sutures, or hemoclips, and partial resection is frequently unsuccessful, especially when thicker than 5 mm. In one Chilean round ray, both epigonal organs were removed during two separate ovariectomy procedures, though the accessory epigonal organ was preserved during the second surgery. Despite bilateral epigonal organ removal, the accessory organ appeared to compensate, as it increased in size ultrasonographically, and no hematologic abnormalities have been noted postoperatively.

Twenty of the 22 cases had complications, with an overall mortality rate of 50%. Anorexia and hyporexia were the most commonly noted post-operative complications, seen in 11 cases, though three of these animals had decreased appetite prior to surgery. Assist feeding was frequently utilized in these cases, along with IV fluids and/or IV Intralipid. The next most common complication was coelomitis (4 cases). Coelomic fluid sampling and flushes were typically utilized to manage cases of coelomitis and effusion, which were often prolonged, lasting weeks to months after surgery. Dehiscence was seen in 4 cases postoperatively. Dehiscence could be due to improper tissue handling, excessive tension on the incision site, or a suture reaction. In severe dehiscence cases, surgical revision was often performed (3 cases), but for more minor cases, close monitoring was elected. In previous reports in healthy southern stingrays, the rate of complications varied, from no major complications other than minor hemorrhage (2) to fatal post-operative coelomitis in 29% post-operatively (7). Therefore, complications are not surprising given advanced disease and likely resulting systemic compromise, further stressing multimodal and perioperative support. In the authors’ experience, rapid intervention after routine monitoring for ovarian disease is preferred to prevent further metabolic decline.

Submission of ovarian tissue for histopathologic evaluation is important to understanding the reproductive disease processes present in elasmobranchs. However, this can be challenging due to inconsistencies in the submitted samples if they are not being evaluated primarily by a pathologist. Ideally, the whole ovary should be submitted; in lieu of that, sampling should include abnormal follicles and at least two different areas of the ovary where the folliculogenic tissues intermingle with the underlying epigonal tissue. There frequently is a discrepancy between clinical impressions and pathology reports: retained, enlarged follicles, while physically abnormal, are not histologically abnormal, and areas of perceived hemorrhage are congested epigonal tissue. Submission of tissues should be accompanied by the gross and clinical data supporting disease, including normal comparisons if available. Specific requests accompanying these submissions, including presence and abundance of atretic or post-ovulatory follicles, size of follicles, and character of the follicular contents (discrete yolk platelets, homogeneous granular material, etc.), can help to build an internal database for institutions and expand our knowledge in this field (K. Conley and C. Rodriguez, personal communication).

This study has a number of limitations, mainly due to the retrospective nature of data collection. As the cases described were treated at six different institutions, the record keeping, case management, diagnostic capabilities, and surgical instrumentation varied, precluding a more standardized review. Additionally, the interpretation of clinical signs and diagnostic imaging may have differed among primary clinicians. Ideally, the ultrasound and computed tomography images would have been evaluated by a single specialist, but this was beyond the scope of this paper.

Ovariectomy represents a viable surgical treatment option for reproductive disease in elasmobranchs, though outcomes may be influenced by species-specific anatomy and physiology, disease severity, and perioperative management. At the time of the writing of this manuscript, there have been two other successful surgeries in elasmobranchs (M. Davis, A. Delaune personal communication). However, dehiscence was noted in one of the cases, and the patient developed coelomitis postoperatively that was managed with periodic flushes. Given the conclusions in this study, refined surgical techniques and careful post-operative management may increase the survival rate (Supplementary material 1). With routine ultrasound or endoscopic monitoring of reproductive anatomy, more rapid surgical intervention can be pursued before the disease results in systemic compromise. Careful species-specific surgical technique and hemostasis are advised, along with intraoperative analgesic, antibiotic, fluid, and metabolic support. Finally, careful monitoring and intensive supportive care are recommended during the post-operative period, given the frequency of post-operative deaths seen in this cohort. The Appendix below should be referenced for more specific pre-surgical, anesthetic, surgical, and post-operative recommendations. With routine monitoring and early surgical intervention, ovariectomy can be a curative treatment for reproductive disease in elasmobranchs.

## 5 Conclusion

- Ovariectomy is a feasible surgical treatment for ovarian and uterine disease in female elasmobranchs and may lead to long-term clinical resolution.
- Ultrasonography is essential for diagnosing reproductive abnormalities and monitoring treatment response in female sharks and rays. Endoscopy and plasma hormone/iodine testing are valuable adjuncts.
- Hormonal and medical management alone were insufficient in resolving disease in this group, emphasizing the importance of surgical intervention in moderate to advanced cases.
- Post-operative complications, including hemorrhage, incision infection, and suture dehiscence, in addition to pre-existing systemic compromise, often led to mortality, emphasizing the need for early intervention and careful perioperative management.

## Data Availability

Publicly available datasets were analyzed in this study. This data can be found here: Veterinary medical records avaialble by FOIA request.
