# Supplementary material for: Surgical management of reproductive disease in aquarium managed female elasmobranchs
Source: Front Vet Sci. 2026 Oct 5;13:1958413. doi: 10.3389/fvets.2026.1958413 (PMC13679300; doi:10.3389/fvets.2026.1958413)
Supplement: Supplementary file 1 [file Supplementary_file_1.docx]

# **Supplementary Material**

**Ovariectomy Planning Checklist**

**Pre-operative Evaluation**

**Imaging**

- Coelomic ultrasound
  - Confirm side, size, and character of ovarian tissue.
  - Evaluate contralateral ovary.
  - Assess uterus (fluid, debris, eggs) and presence of coelomic effusion.
  - Assess liver and body condition to determine post-operative nutritional management.
- Endoscopy (if available):
  - Cloacoscopy/hysteroscopy – endometrium, uterine contents, sampling.
  - Coelioscopy – ovary, epigonal organ, liver, coelomic fluid.

**Laboratory testing**

- CBC and plasma biochemistry, including PCV/hematocrit.
- Coelomic fluid cytology and culture as a baseline.
- Plasma estradiol and total iodine.

**Anesthetic Planning and Monitoring**

**Induction & maintenance**

- Induction: buffered MS-222 (approx. 60–100 ppm) or eugenol (approx. 30–60ppm) formulations at species-appropriate doses.
- Maintenance: MS-222 (approx. 50–80 ppm) with ability to titrate down for longer procedures.
- Consider IV butorphanol as an adjunct. Doses of 0.1-0.5 mg/kg were utilized in this cohort

**Set-up**

- Anesthetic table with drainage and padding (foam, bubble wrap, soft cloth or flannel, towels).
- Continuous gill irrigation with oxygenated anesthetic water via Y-piece.
- Maintain high dissolved oxygen (10-12 mg/dL).

**Monitoring**

- Cardiac ultrasound or Doppler for heart rate, rhythm, and contractility.
- Periodic blood gases, lactate, ionized calcium.

**Support**

- IV fluids (e.g., Elasmobranch Ringer’s).
- Intralipid IV as needed.
- Heparinized allogenic blood transfusion if significant hemorrhage occurs.

**Surgical Planning and Technique**

**Positioning & environment**

- Position on padded table; maintain skin and eye moisture.
- Clear drapes, if used, to allow visualization of ventilatory movements.

**Skin preparation**

- Gently remove biofilm with saline only or dilute antiseptic; avoid vigorous scrubbing.
- Use minimal disinfectant – some individuals react to products like povidone-iodine.

**Incision placement**

- Myliobatiformes: dorsal left (or right if needed) paramedian/paralumbar incision.
- Orectolobiformes, Charcarhiniformes: right dorso-lateral or flank incision caudal to the pectoral fin.

**Handling of diseased tissue**

- Incisional handling and manipulation should be limited and avoid grabbing instruments where possible.
- Expect large, friable, highly vascular ovaries/uteri.
- Use gentle traction or stay sutures instead of heavy instrument grasping.
- Aspiration of large cysts can improve visualization and facilitate removal.
- Sectioned removal of very large ovaries can be considered.

**Epigonal organ**

- Often intimately associated with the ovary; separation can be difficult and risky due to hemorrhage. It is easy to incidentally leave functional gonadal tissue with epigonal remnants.
- *En bloc* removal with the ovary is acceptable and often preferable when safe separation is not possible.
- Take care near major vessels (e.g., caudal vena cava); vessel-sealing devices and cautery are highly useful.

**Uterus**

- Uterine fluid may need to be removed prior to surgery
- Hysterectomy is not recommended
  - Removal of the uterus is unlikely to resolve hormonally driven diseases
  - Hysterectomy is a technically difficult surgery due to difficult hemostasis
  - Due to the proximity of the kidneys, hysterectomy may inadvertently or directly damage renal structures.

**Hemostasis**

- Vessel-sealing device strongly recommended for ovarian connective tissue, epigonal tissue, and vascular pedicles.
  - Epigonal tissue >5mm should not be transected, identify thinner areas for removal, or remove completely.
- Monopolar/bipolar cautery for smaller vessels and diffuse bleeding.
- Monitor intra-operative blood loss and hematocrit; be prepared to transfuse.
- Gel foam does not appear to be absorbed.

**Coelomic assessment**

- Assess the coelom for other obvious signs of disease, especially liver health. If the liver appears small or in poor condition, more intensive supportive care may be necessary postoperatively.
- Perform a complete exploration of the coelom, as a uterine rupture may lead to free-floating egg cases or ovarian follicles may have fragmented.

**Closure and Suture Strategy**

**Layered closure**

- 2 layers:
  - Body wall (primary holding layer)
  - Muscle/fascia (optional, often weak)
  - Skin (major external holding layer)
- Use tension-relieving patterns (horizontal/vertical mattress, cruciate), minimizing the number and bulk of external knots.

**Suture material**

- Absorbable sutures often persist for many years.
- External reactions are subjectively more common with some materials (e.g., polydioxanone).
- Consider monofilament absorbable sutures (e.g., poliglecaprone), though it should be noted that absorbable sutures often do not dissolve internally
- Use long curved needles to accommodate body wall thickness.

**Dehiscence prevention**

- Rinse sutures pre-use to reduce potential irritants.
- Leave sutures in longer than in teleost or terrestrial species, as incisions may appear healed then fail late. Sutures should not be removed until at least 6 weeks postoperatively.
  - Based on the authors’ experience, if coelomitis is present, sutures should not be removed until coelomitis is resolved.

**Peri-Operative Supportive Care**

**Fluid & Metabolic Support**

- Elasmobranch Ringer’s (37)
- Intralipid (1-5 ml/kg used in this cohort)
- If excessive intraoperative hemorrhage:
  - Blood transfusion (38)
  - Epogen (200 IU/kg IM)

**Analgesia and Antibiotics**

- Intraoperative and post-operative NSAIDs.
- Local analgesia (bupivacaine, lidocaine) can be considered as a splash block.
- Intra-operative and post-operative antibiotics, ideally based on culture and sensitivity results.

**Ophthalmic Protection**

- Eye lubrication throughout the procedure.
- Fluorescein stain after surgery to rule out corneal trauma.

**Post-Operative**

**Supportive Care**

- Assist feeding, fluid therapy with Intralipid, and continued antibiotic/analgesic therapy as necessary.
- Copious coelomic flushing with sterile saline or Elasmobranch Ringer’s +/- antibiotics through a coelomic pore is frequently necessary with chronic coelomitis.

**Histopathology**

- Submission of the full removed ovary and epigonal organ for histopathologic evaluation with multiple representative sections sampled.

**References**

37.	MyIniczenko ND, Clauss T. "Pharmacology of elasmobranchs: updates and techniques". In: Smith M, Warmolts D, Thoney D, Hueter R, Murray M, Ezcurra J, editors. Elasmobranch Husbandry Manual II: Recent Advances in the Care of Sharks, Rays, and their Relatives. Columbus, Ohio: Ohio Biological Survey. (2017). p. 289–302.

38.	Delaune AJM, Field CL, Clauss TM. Whole blood transfusions to treat severe anemia in seven cownose rays (*Rhinoptera bonasus*) and one short-tail stingray (*Dasyatis brevicaudata*). J Zoo Wildl Med. (2017) 48(4):1172–80.
